# Incidence of and Risk Factors for Complex Regional Pain Syndrome Type 1 after Surgery for Distal Radius Fractures: A Population-based Study

**DOI:** 10.1038/s41598-019-41152-x

**Published:** 2019-03-19

**Authors:** Young-Hoon Jo, KangWook Kim, Bong-Gun Lee, Joo-Hak Kim, Chang-Hun Lee, Kwang-Hyun Lee

**Affiliations:** 1 Department of Orthopedic Surgery, Sungmin Hospital, Incheon, 22789 South Korea; 2 Department of Orthopedic Surgery, Seouldaejung Hospital, Cheonan, 31156 South Korea; 3 Department of Orthopedic Surgery, Hanyang University College of Medicine, Seoul, 04763 South Korea; 4 Department of Orthopedic Surgery, Myongji Hospital, Goyang, 10475 South Korea; 5 Department of Orthopedic Surgery, Eulji University College of Medicine, Seoul, 01830 South Korea

## Abstract

This study aimed to evaluate the incidence rates of and risk factors for complex regional pain syndrome type 1 (CRPS-1) after surgery for distal radius fractures (DRFs). Using data from January 2007 to December 2014, we analysed the data from the Korean Health Insurance Review and Assessment (HIRA) service. After extracting the data of patients aged ≥18 years whose diagnostic and operation codes for DRFs were entered into the HIRA database, we analysed the incidence rates of and risk factors for CRPS-1. From 2007 to 2014, 172,194 DRFs were treated surgically. Within 1 year postoperatively, 1,103 CRPS-1 cases were diagnosed, with an incidence of 0.64%. On univariate and multivariate analyses, the risk factors that significantly correlated with the incidence of CRPS-1 included female sex, rheumatoid arthritis, open reduction, open fracture, and accompanying ulnar fracture, whereas old age, psychiatric disease, and external fixation were not statistically significant. The incidence of CRPS-1 after surgery for DRF was very low (0.64%) in South Korea. Careful monitoring is necessary for patients with complex fractures and rheumatoid arthritis who are at increased risk of developing CRPS-1.

## Introduction

Complex regional pain syndrome type 1 (CRPS-1) is a complex clinical syndrome that is characterised by excessive pain, swelling, local blood flow changes, and abnormality of the sudomotor nerve. It occurs frequently in the upper extremities and is often seen after fractures or surgery^1^. Therefore, distal radius fractures (DRFs) are one of the most common causes of CRPS-1. When CRPS-1 occurs, the patient’s quality of life and daily function are severely impaired due to the severe pain, making it difficult for them to return to the workplace^2^. Early diagnosis and intensive treatment of CRPS-1 are important for recovery of function^2,3^. Therefore, it is important to recognize the risk factors for CRPS-1 to enable early diagnosis and intervention.

Most previous studies covered a small number of patients at single institutions, and there was no consensus on the diagnostic criteria for CRPS-1. Therefore, the incidence of CRPS-1 after DRF was significantly different amongst the studies, ranging from 0.9% to 37%^4–7^. In addition, several studies have suggested that various risk factors are associated with the occurrence of CRPS-1, but there is disagreement amongst them on the relationship between these risk factors and the incidence of CRPS-1^8–10^. Some studies have suggested that psychiatric disorders, such as depression and anxiety disorders, cause CRPS-1^11,12^. In other studies, psychiatric disorders were not a trigger but a result of chronic pain and disability^13,14^. Studies have suggested that treating DRFs with external fixation (EF) increases the incidence of CRPS-1 compared with other surgical methods because of its long fixation period and distraction technique^15,16^, whereas other studies have suggested that there is no significant difference in the incidence of CRPS-1 depending on the surgical method^17,18^. Additionally, several studies have suggested that the incidence of CRPS-1 is correlated with old age, female sex, rheumatoid arthritis (RA), and fracture types; however, there is no clear consensus^8,10^.

We evaluated the incidence of CRPS-1 in patients who underwent surgical treatment of DRF, using the nationwide database of the Korean Health Insurance Review and Assessment (HIRA) service. We also investigated the relationship between the incidence of CRPS-1 and risk factors such as age, sex, concomitant diseases, type of surgery, and type of fracture.

## Results

From 2007 to 2014, 172,194 DRFs (157,398 patients) were treated surgically. Of these, 34,875 patients (37,929 DRFs) were male and 122,523 (134,265 DRFs) were female. The mean age was 61.7 ± 13.8 years. CRPS-1 occurred after surgery for DRF in 1,103 cases; the incidence rate was 0.64%. The time at which the International Classification of Diseases, 10th revision (ICD-10) code for CRPS-1 was entered was 97 ± 84 days from surgery. The incidence was lowest in patients in the under-30s group (0.24%) and steadily increased until patients were in their 50 s (0.83%). Afterwards, the incidence declined again and showed a low rate (0.30%) at the age of 80 (Table 1). In terms of the surgical method, the incidence was highest (0.88%) when open reduction and internal fixation (ORIF) and EF were simultaneously performed and lowest (0.56%) when closed reduction and percutaneous pinning were performed. The incidence of CRPS-1 was lower after EF (0.61%) than after ORIF (0.68%) (Table 2).

**Table 1:** The incidence of type 1 CRPS by age group.

Age (year)	Total	Men	Women
	No of CRPS patient/No of fracture patient	No of CRPS patient/No of fracture patient	No of CRPS patient/No of fracture patient
18–29	14/5,752 (0.24%)	9/4,275 (0.21%)	5/1,477 (0.34%)
30–39	18/5,815 (0.31%)	14/3,942 (0.36%)	4/1,873 (0.21%)
40–49	70/14,754 (0.47%)	30/7,777 (0.39%)	40/6,977 (0.57)
50–59	374/45,319 (0.83%)	57/9,813 (0.58%)	317/35,506 (0.89%)
60–69	359/47,771 (0.75%)	51/6,681 (0.76%)	308/41,090 (0.75%)
70–79	226/38,945 (0.58%)	24/4,319 (0.55%)	202/34,626 (0.58%)
80+	42/13,838 (0.30%)	0/1,122 (0%)	42/12,716 (0.33%)

CRPS: complex regional pain syndrome.

**Table 2:** The incidence of Type 1 CRPS by surgical method.

Surgical methods	CRPS cases/Total cases	Incidence rate
Open reduction and internal fixation	517/75,230	0.68%
Closed percutaneous pinning	324/57,272	0.56%
Open reduction and internal fixation with External fixation	19/2,129	0.88%
Closed percutaneous pinning with External fixation	70/8,464	0.82%
External fixation	173/27,996	0.61%

CRPS: complex regional pain syndrome.

In the univariate analysis, patients in the CRPS group were significantly older than those in the non-CRPS group, and there was a higher ratio of women (all: p < 0.001). The proportion of patients with psychiatric factors and RA was significantly higher in the CRPS group than in the non-CRPS group (p = 0.044 and p = 0.001, respectively). Regarding the fracture type, patients in the CRPS group had a significantly greater incidence of accompanying ulnar fracture and open fractures (p = 0.001 and p = 0.007, respectively) than those in the non-CRPS group. Regarding surgical methods, although the proportion of cases in which open reduction was performed was higher in the CRPS group than in the non-CRPS group (p = 0.024), there was no significant difference in the proportion of EFs between the two groups (p = 0.342) (Table 3).

**Table 3:** Univariate analysis of the CRPS group and non-CRPS group.

Variable	CRPS group (n = 1,103)	Non-CRPS group (n = 171,091)	P-value
Sex, n (%)			<0.001
Male	185 (16.8)	37,744 (22.1)	.
Female	918 (83.2)	133,347 (77.9)	.
Age, years	61.84 ± 10.94	61.67 ± 13.86	<0.001
Psychiatric factor, n (%)	138 (12.5)	18,201 (10.6)	0.044
Seropositive RA, n (%)	22 (2.0)	1,514 (0.9)	<0.001
Reduction method, n (%)			0.024
Open	536 (48.6)	77,359 (45.2)	
Closed	567 (51.4)	93,732 (54.8)	
Application of external fixator, n (%)	262 (23.8)	38,589 (22.6)	0.342
With ulnar fracture, n (%)	386 (35.0)	47,366 (27.7)	<0.001
Open fracture, n (%)	55 (5.0)	5,984 (3.5)	0.007

CRPS: complex regional pain syndrome; RA: rheumatoid arthritis.

In the multivariate logistic regression analysis, female sex, RA, accompanying ulnar fracture, open fracture and open reduction were significant factors, whereas age and psychiatric factors were not (p = 0.077 and p = 0.088, respectively). Amongst the significant factors, RA had the highest odds ratio, followed by female sex and accompanying ulnar fracture (Table 4).

**Table 4:** Multivariate logistic regression for predicting type 1 complex regional pain syndrome after distal radius fracture surgery.

Variable	aOR	95% CI	P-value
Sex	1.448	1.223–1.723	<0.001
Age, years	1.043	0.995–1.094	0.077
Psychiatric factor	1.170	0,973–1.396	0.088
Seropositive RA	2.185	1.385–3.258	<0.001
Reduction method	1.148	1.019–1.293	0.023
With ulnar fracture	1.376	1.213–1.559	<0.001
Open fracture	1.338	1.004–1.746	0.039

aOR: adjusted odds ratio; CI: confidence interval; RA: rheumatoid arthritis.

## Discussion

Recently, the preference for surgical treatment of DRF has increased^19,20^, and some studies have linked the high incidence of CRPS-1 with EF^15,16^. Thus, we analysed the incidence rate of and risk factors for CRPS-1 in 172,194 DRFs treated surgically from 2007 to 2014 using the nationwide database of the Korean HIRA. The incidence of CRPS-1 was 0.64%, which was lower than that reported previously. Female sex, RA, accompanying ulnar fracture, open fracture, and open reduction were identified as significant risk factors for CRPS-1, whereas age, psychiatric factors, and EF were not.

Kim *et al*.^21^ reported the incidence of CRPS in South Korea as 29.0 per 100,000 person-years; this rate is higher than that in western countries, where it is 26.2 per 100,000 person-years in the Netherlands and 5.46 per 100,000 person-years in the US^1,22^. Therefore, the low incidence rate of CRPS after surgery for DRFs in this study may not be due to ethnic differences. In South Korea, CRPS-1 has been classified as an intractable and rare disease since 2005 and has become subject to the Copayment Decreasing Policy. If the ICD-10 code for CRPS-1 is entered and the Copayment Decreasing Policy application is submitted to and accepted by the National Health Insurance (NHI) corporation, the patient will only need to pay 10% of the total medical cost. When fully developed CRPS-1 that requires treatment occurs, the surgeon or physician should enter ICD-10 codes based on the diagnostic criteria to reduce the patient’s economic burden. Based on the results of our study, fully developed CRPS-1 requiring treatment after surgery for DRF appears to be rare. In the United States, Crijns *et al*.^23^ also analysed the incidence rate of CRPS after DRFs using the Truven Health MarketScan database and reported a low incidence rate of 0.19%.

Several studies have reported that the incidence of CRPS-1 after EF for DRF was higher than that after other types of surgery^15,16^. However, EF was not a significant risk factor for CRPS-1 in this study. When the incidence of CRPS-1 was analysed according to the surgical method, the incidence was lower when only EF was performed than it was when ORIF was performed. In the treatment of DRFs, EF is indicated when there is a severe intra-articular comminuted fracture or complex dislocation of the carpus^24^. Therefore, EF is usually performed for severe DRFs. A previous study suggested that the high incidence of CRPS-1 is not caused by EF, but rather due to the severe injury^15^. Jo *et al*.^19^ reported on the surgical trends in DRF treatment at different types of hospitals in South Korea. In Korea, tertiary hospitals (3.1%) had a lower rate of EF procedures than general hospitals (23.3%) and small hospitals (21.3%). Considering that severely injured patients are highly likely to be transferred to tertiary hospitals, which are more advanced health-care institutions than general and small hospitals, in Korea, it seems that EF was indicated not only for severe injuries, but also factors such as the surgeon’s preference and economic aspects. Because of this background, EF was not identified as a risk factor for the development of CRPS-1 in this study. We believe that the incidence of CRPS-1 will not increase if EF is performed in patients with simple fractures.

There is significant controversy regarding the relationship between psychiatric factors and CRPS-1^9,25^. Some studies reported a high incidence of CRPS-1 amongst patients with psychiatric factors, such as anxiety disorder and depression, whereas others argued that there is no clear relationship between them^12,14,25^. In this study, these psychiatric factors were not significant risk factors for CRPS-1. It is known that histological changes occur around CRPS-1 lesions^26^. Zollinger *et al*.^5^ have argued that it is difficult to explain the changes and abnormalities in the tissues by psychiatric factors only.

Some studies have suggested that CRPS-1 occurs in elderly people^10,25^. However, age was not a significant risk factor in this study. The incidence of CRPS-1 increased with age until the patients were in their 50 s, but decreased thereafter (Table 1). In previous population-based studies, a similar pattern of incidence by age group was observed^1,22^. Based on this result, it appears that the incidence of CRPS-1 does not simply with age in a linear fashion, but that there are age groups in which CRPS-1 occurs most frequently. The incidence of CRPS-1, as well as the number of patients with DRF, was higher amongst patients in their 50 s and 60 s than amongst patients in any other age group.

Female sex has been reported as a risk factor for CRPS-1^1,10,22^, and it was also a significant risk factor in this study. Although the incidence of surgery for DRF was 3.5 times higher amongst women than that amongst men, the incidence of CRPS-1 was five times higher amongst women than that amongst men (Table 3). Because the incidence of CRPS-1 is especially high amongst women in their 50 s and 60 s, CRPS-1 may be a result of hormonal changes; however, the relationship between CRPS-1 and hormones is still unclear^27^.

Interestingly, seropositive RA was a significant risk factor for CRPS-1, with the highest odds ratio (Table 4). Several authors have suggested that an inflammatory response is a major mechanism of CRPS-1^28,29^. The levels of neuropeptides, such as substance P and the calcitonin gene-related peptide, are increased in patients with CRPS-1^30,31^, and these neuropeptides cause vasodilation and protein extravasation, resulting in neurogenic inflammation with erythema, oedema and heat^32^. The serum levels of inflammatory cytokines, such as tumour necrosis factor α and interleukin 6, are also elevated in patients with CRPS-1^29,33^, and these cytokines are known as major causes of sensitisation and hyperalgesia^34,35^. Based on these findings, neurogenic inflammation plays an important role in the pathogenesis of CRPS-1^28^.

Various inflammatory cytokines and neuropeptides are involved in the pathogenesis of RA^36^. The levels of cytokines and neuropeptides in the serum and synovial fluid of patients with RA are high, and elevated levels are associated with disease activation^37–39^. Furthermore, because the wrist joints are the most common sites of RA lesions^40,41^, DRFs can be exposed to high levels of cytokines and neuropeptides that are present in the synovial fluid of the wrists of patients with RA^39^. These cytokines and neuropeptides may induce neurogenic inflammation and vasomotor dysfunction; therefore, RA may be a risk factor for CRPS-1. Beerthuizen *et al*.^8^ also reported that RA is a risk factor for CRPS-1.

Accompanying ulnar facture and open fractures were significant risk factors for CRPS-1. Lafontaine *et al*.^42^ classified DRFs that were associated with an ulnar fracture as unstable fractures in patients older than 60 years. Bicherstaff and Kanis^43^ reported that the incidence of CRPS-1 after DRF was high when it was accompanied by an ulnar fracture, and the authors reported that the high incidence of CRPS-1 was associated with the severity of the fracture. In addition, open fractures are more often caused by soft tissue damage and high-energy injury than closed fractures^44^. Several studies have reported on the incidence of CRPS-1 after severe injury^5,8,10,17^. It appears that accompanying ulnar fracture and open fracture are risk factors for CRPS-1 and are associated with the severity of injury. In this study, the incidence of CRPS-1 was the highest when ORIF and EF were performed simultaneously (Table 2). This result also demonstrates the relationship between severe complex fractures and CRPS-1.

ORIF has become the standard surgical method for DRF worldwide^19,20,45^. In this study, the incidence of CRPS-1 was higher after open reduction than after closed reduction. This result is not surprising because any surgery that damages the soft tissues can cause CRPS-1^10,46^. Although open reduction had the lowest odds ratio, a precise surgical technique that minimises soft tissue damage during open reduction must be developed to reduce the incidence of CRPS-1.

The main strength of the present study was the inclusion of a large number of patients using a nationwide database. Because CRPS-1 is a relatively rare disease, there is a considerable limitation in analysing risk factors from a single institution^4^. In addition, the diagnostic criteria of CRPS-1 were uniformly applied using national criteria. However, there are limitations to this study as well. First, the diagnostic criteria for entering the ICD-10 codes of CRPS-1 in South Korea during the study period were the original International Association for the Study of Pain (IASP) criteria rather than the new IASP criteria (also called the Budapest criteria)^47,48^. Although the original IASP criteria have a lower specificity than the Budapest criteria^49^, in South Korea, unfortunately these were the only diagnostic criteria accepted by the NHI during the study period. Second, patients who underwent conservative treatment with DRFs were not included in the study. The authors wanted to analyse if there was any difference in the incidence rate of CRPS according to the surgical methods for DRFs, specifically whether EF is an important risk factor for CRPS. Hence, the authors only included patients who underwent surgical treatment after DRFs in this study. Third, there was no radiographic information on DRF. Finally, there may have been some coding errors in this large database.

In conclusion, the incidence of CRPS-1 after surgery for DRF was very low (0.64%) in South Korea. Furthermore, careful monitoring is necessary for patients with complex fractures and RA, who are at increased risk of developing CRPS-1. If CRPS-1 is suspected during patient monitoring, early intervention with proactive anti-inflammatory therapy, such as steroids, may be helpful.

## Methods

### Data source

The authors analysed a nationwide database obtained from the HIRA from 2007 to 2014. In South Korea, NHI covers 100% of the population, including 97% of health insurance and 3% of medical aid^19^. All health-care providers submit claims data for inpatient and outpatient management, including diagnostic codes, which are classified according to the ICD-10 codes, procedure codes and demographic information, to the HIRA to request reimbursement for the medical cost from the NHI service. Hence, the medical records of almost all outpatients or hospitalised patients at health-care institutions in South Korea are prospectively recorded in the HIRA database.

### Data collection

We first identified patients aged ≥18 years who underwent surgery for DRF from 2007 to 2014, using ICD-10 codes (S525 and S526) and operation codes (Table 5)^19^. The presence of CRPS-1 was assessed using the ICD-10 codes for CRPS-1 (M890). The diagnostic criteria of CRPS-1 in South Korea are based on the criteria of the IASP^47^, and the CRPS pattern that appears on a three-phase bone scan or infrared thermography should show a difference of greater than 1° compared with that of the normal side. In this study, CRPS-1 that was associated with DRF was defined as CRPS-1 that occurred within 1 year from surgery for DRF. The ICD-10 code for CRPS-1 (M8905, M8906, M8097, and M8908) in the lower extremities was considered to be independent from DRF.

**Table 5:** Operation codes of distal radius fractures in South Korea.

Operation Code	Description
N0607 or N0617	ORIF for radius
N0603 or N0613	ORIF for both radius and ulna
N0993	Percutaneous pinning for radius
N0994	Percutaneous pinning for radius and ulna
N0983	External fixation

ORIF: open reduction with internal fixation.

HIRA data from 2007 to 2015 were available, but it was difficult to confirm the occurrence of CRPS-1 in patients with DRFs in 2015 because of the short follow-up period. The 2015 data were only used to confirm the occurrence of CRPS-1 in patients with DRFs in 2014. In this study, patients younger than 18 years, patients who received a diagnostic code for CRPS-1 before DRF and patients who received a diagnostic code for CRPS-1 more than 1 year after DRF were excluded.

We examined the patients’ age, sex, comorbidities, operative method and fracture type. Psychiatric disorders and RA, which were shown to be associated with CRPS-1 in previous studies, were examined as comorbidities^8,25^. Because the HIRA data provide information on the medical specialty responsible for treatment, patients who were diagnosed with depression or anxiety disorder by a psychiatrist prior to DRF were determined to have psychiatric factors (Table 6)^9^. The presence of RA was determined by the presence of ICD-10 codes for seropositive RA (Table 6). The operative method was determined by using the operation code to investigate whether open reduction or EF was performed (Table 5). Due to the lack of radiological information on DRFs in the HIRA data, the presence of a distal ulnar fracture (S526) and open fracture (S52521, S52531, S52541, S52581, S52591, and S5261) were investigated as the fracture type, using the ICD-10 codes.

**Table 6:** Diagnosis codes of depression, anxiety disorder and seropositive rheumatoid arthritis according to the ICD-10.

Comorbidity	ICD-10 Code	Description
Depression	F32	Depressive episode
	F33	Recurrent depressive disorder
	F341	Dysthymia
	F381	Recurrent brief depressive episodes
	F412	Mixed anxiety and depressive disorder
	F920	Depressive conduct disorder
Anxiety disorder	F40	Phobic anxiety disorders
	F41	Other anxiety disorders
	F064	Organic anxiety disorder
Seropositive RA	M058	Other seropositive RA
	M059	Seropositive RA, unspecified

ICD-10: International Classification of Diseases, 10th revision; RA: rheumatoid arthritis.

### Statistical analysis

The patients were divided into two groups: patients with CRPS-1 (CRPS group) and those without CRPS-1 (non-CRPS group) to investigate the risk factors for CRPS-1. Variables in the two groups were compared using Student’s t-tests or the chi-square test. A multivariate logistic regression analysis was used to examine factors affecting the occurrence of CRPS-1, considering the confounding effect of variables showing significance in the univariate analysis. A p-value < 0.05 was considered statistically significant. The statistical analysis was performed using the SAS statistical software, version 9.13 (SAS Institute, Cary, NC, USA).

### Ethics approval and consent to participate

This study protocol was exempted for review by the institutional review board of the Hanyang University Hospital (HYUH 2016-05-003) in accordance with the exemption criteria. Informed consent was exempted because this study used only data opened to the public.

## Data Availability

Data will not be shared due terms of the contract signed with the Korean Health Insurance Review and Assessment Service that provided us with the nationwide data in South Korea. According to these terms, the data collected must be discarded once the investigation has been concluded.
